# Tropine exacerbates the ventilatory depressant actions of fentanyl in freely-moving rats

**DOI:** 10.3389/fphar.2024.1405461

**Published:** 2024-06-24

**Authors:** Paulina M. Getsy, Walter J. May, Alex P. Young, Santhosh M. Baby, Gregory A. Coffee, James N. Bates, Yee-Hsee Hsieh, Stephen J. Lewis

**Affiliations:** ^1^ Department of Pediatrics, Case Western Reserve University, Cleveland, OH, United States; ^2^ Department of Pediatrics, University of Virginia, Charlottesville, VA, United States; ^3^ Galleon Pharmaceuticals, Inc., Horsham, PA, United States; ^4^ Department of Anesthesiology, University of Iowa Hospitals and Clinics Iowa, Iowa City, IA, United States; ^5^ Division of Pulmonary, Critical Care and Sleep Medicine, University Hospitals Case Medical Center, Case Western Reserve University, Cleveland, OH, United States; ^6^ Department of Pharmacology, Case Western Reserve University, Cleveland, OH, United States; ^7^ Functional Electrical Stimulation Center, Case Western Reserve University, Cleveland, OH, United States

**Keywords:** tropine, fentanyl, ventilatory depression, arterial blood-gas chemistry, analgesia, male Sprague Dawley rats

## Abstract

Our lab is investigating the efficacy profiles of tropine analogs against opioid-induced respiratory depression. The companion manuscript reports that the cell-permeant tropeine, tropine ester (Ibutropin), produces a rapid and sustained reversal of the deleterious actions of fentanyl on breathing, alveolar-arterial (A-a) gradient (i.e., index of alveolar gas exchange), and arterial blood-gas (ABG) chemistry in freely-moving male Sprague Dawley rats, while not compromising fentanyl analgesia. We report here that in contrast to Ibutropin, the injection of the parent molecule, tropine (200 μmol/kg, IV), worsens the adverse actions of fentanyl (75 μg/kg, IV) on ventilatory parameters (e.g., frequency of breathing, tidal volume, minute ventilation, peak inspiratory and expiratory flows, and inspiratory and expiratory drives), A-a gradient, ABG chemistry (e.g., pH, pCO_2_, pO_2_, and sO_2_), and sedation (i.e., the righting reflex), while not affecting fentanyl antinociception (i.e., the tail-flick latency) in freely-moving male Sprague Dawley rats. These data suggest that tropine augments opioid receptor-induced signaling events that mediate the actions of fentanyl on breathing and alveolar gas exchange. The opposite effects of Ibutropin and tropine may result from the ability of Ibutropin to readily enter peripheral and central cells. Of direct relevance is that tropine, resulting from the hydrolysis of Ibutropin, would combat the Ibutropin-induced reversal of the adverse effects of fentanyl. Because numerous drug classes, such as cocaine, atropine, and neuromuscular blocking drugs contain a tropine moiety, it is possible that their hydrolysis to tropine has unexpected/unintended consequences. Indeed, others have found that tropine exerts the same behavioral profile as cocaine upon central administration. Together, these data add valuable information about the pharmacological properties of tropine.

## Introduction

Tropine ring structures (Trautner and McCallum, 1950; Zeile and Heusner, 1959; Issekutz, 1963) are vital components of many bioactive drugs, such as cocaine, atropine, and a variety of neuromuscular blockers, such as decamethylene bis N,N′ atropinium and bis [N (3,4-diacetoxybenzyl) tropaninium (alpha-yl) glutarate] dibromide (Gyermek, 2002; Kohnen-Johannsen and Kayser, 2019). Our companion manuscript (Getsy et al., 2024) details evidence that intravenous injection of the cell-permeant tropeine, tropine ester (Ibutropin), also known as isobutyric tropine ester; butropine; tropine isobutyrate, iisobutyroyl tropine, elicits a rapid and sustained reversal of the adverse actions of fentanyl on breathing, alveolar-arterial (A-a) gradient (i.e., index of alveolar gas exchange), and arterial blood-gas (ABG) chemistry (i.e., pH, pCO_2_, pO_2,_ and sO_2_), while not reducing fentanyl-induced analgesia in unanesthetized male Sprague Dawley rats. Although we do not have direct information about Ibutropin, we expect that it will be highly cell-penetrant like other tropeines (Gyermek, 2002; Kohnen-Johannsen and Kayser, 2019). The actions of Ibutropin are likely to involve effects on functional proteins in the plasma membrane (e.g., ion-channels and receptors) and functional proteins within cells. Previous research has shown that tropeines exert direct effects on serotonin, acetylcholine, and histamine receptor subtypes (Gyermek, 1953a; b, Gyermek, 1953, 2002; Zaĭtseva and Gerchikov, 1969; Mashkovskiĭ and Shvarts, 1979; Gyermek and Lee, 2009a; b), and allosterically regulate glycine ion-channel receptor activity (Macksay et al., 2004; 2008; 2009a; b; San Martin et al., 2019) along with the activity of other ion channels, such as Na^+^ and K^+^-channels (Friess et al., 1961a; b; 1963; 1964a; b, 1965a; b; 1966; 1968; 1969; Blaustein, 1968; Thron et al., 1963). All the receptors and ion-channels mentioned above have important roles in the central regulation of breathing (Richter et al., 2003; Shao and Feldman, 2009; Manzke et al., 2011). Nonetheless, to date, no information has been reported as to whether tropine has any effects on ventilatory control systems.

At present, there is little direct information about the actual metabolites of Ibutropin or their pharmacological actions *in vivo*. However, the potential desterification of Ibutropin to tropine (Supplementary Figure S1) by nonspecific carboxyesterases within blood plasma (Butterworth et al., 1993; Nishida et al., 1996; Hemmings and Egan, 2018) may be a factor in the ability of Ibutropin to exert its potent effects against opioid-induced respiratory depression (OIRD) (Getsy et al., 2024). This is especially possible because tropine exerts the same behavioral profile as cocaine upon central administration to rats (Zakusov et al., 1978). As such, the objectives of this study were to determine the effects an injection of tropine (200 μmol/kg, IV) has on the deleterious actions of fentanyl (75 μg/kg, IV) on breathing by looking at changes in ventilatory parameters, A-a gradient, ABG chemistry, antinociception and sedation (i.e., righting reflex) in freely-moving (unanesthetized) adult male Sprague Dawley rats. The data collected in this study provides evidence that in contrast to Ibutropin, the administration of tropine largely exacerbates the opioid receptor signaling events that mediate the effects of fentanyl on breathing, alveolar gas exchange, and sedation, while not affecting fentanyl-induced antinociception. Therefore tropine, when liberated by the hydrolysis of Ibutropin, appears to combat the Ibutropin-induced reversal of the adverse effects of fentanyl on breathing. Whether the ability of tropine to exacerbate the effects of fentanyl is due to allosteric modulation of opioid receptors and/or modulation of intracellular cascades elicited by fentanyl will be the focus of future studies.

## Material and methods

### Permissions, rats, and surgical procedures

All studies were carried out in accordance with the NIH Guide for Care and Use of Laboratory Animals (NIH Publication No. 80-23) revised in 2011, and in compliance with the ARRIVE (Animal Research: Reporting of *In Vivo* Experiments) guidelines (https://arriveguidelines.org/). All protocols involving rats were approved by the Animal Care and Use Committees of Galleon Pharmaceuticals, Case Western Reserve University, and the University of Virginia. Adult male Sprague Dawley rats were purchased from *Harlan Industries* (Madison, WI, United States). After 4 days of recovery from transportation, rats were implanted with a jugular vein catheter only or with a jugular vein catheter and a femoral artery catheter under 2%–3% isoflurane anesthesia (Henderson et al., 2014; Gaston et al., 2021). The rats were given 4 days to recover from surgery before use in any experiment. All catheters were flushed with a heparin solution (50 units of heparin in 0.1 M, pH 7.4 phosphate-buffered saline) immediately after surgery and again 8 h later. The catheters were also flushed twice-daily at 8 a.m. and 4 p.m. on recovery days 2–4 and again 3–4 h before starting a study on post-surgery day 5 (therefore, nine flushes in total). Injectable (liquid) fentanyl citrate and tropine powder were obtained from *Sigma-Aldrich* (St. Louis, MO, United states). The pH of all stock solutions of vehicle and tropine was adjusted to 7.0 with 0.25 M NaOH. All studies were performed in a quiet room with a relative humidity of 49% ± 2% and room temperature of 21.3°C ± 0.2°C. The ABG chemistry and antinociception studies were done in separate groups of rats so as not to compromise the ventilatory recording studies. The plethysmography, antinociception recording sessions and arterial blood sampling studies (used for ABG measurements) were performed by an investigator who was blinded to the study protocol and thus used syringes with the opioid, vehicle or test drugs that were prepared by another investigator who was not involved in the study protocol. In every case, the data files resulting from each study were collated and analyzed by yet another investigator in the group who did not make up the syringes or perform the study protocol.

### Whole body plethysmography measurement of ventilatory parameters

Ventilatory parameters were recorded continuously in unrestrained, freely-moving rats by whole body plethysmography (PLY3223; *Data Sciences International*, St. Paul, MN), as detailed previously (Getsy et al., 2022a; b). The directly recorded and calculated (derived) parameters are defined in Supplementary Table S1. The ventilatory parameters and abbreviations are frequency of breathing (Freq), tidal volume (TV), minute ventilation (MV), inspiratory time (Ti), expiratory time (Te), Ti/Te, end inspiratory pause (EIP), end expiratory pause (EEP), peak inspiratory flow (PIF), peak expiratory flow (PEF), PIF/PEF, expiratory flow at 50% expired TV (EF_50_), relaxation time (RT), inspiratory drive (TV/Ti), expiratory drive (TV/Te), expiratory delay (Te-RT), non-eupneic breathing index (NEBI), and NEBI corrected for Freq (NEBI/Freq). A diagram, adapted from Lomask (2006), showing relationships between some directly recorded parameters is shown in Supplementary Figure S2. On the day of the study, each rat was placed in an individual plethysmography chamber and allowed at least 60 min to acclimatize so that resting (baseline, Pre) ventilatory parameter values could be accurately defined. Two groups of rats (see Supplementary Table S2 for numbers, ages, weights, and baseline ventilatory parameters) received an injection of fentanyl (75 μg/kg, IV). After 5 min, one group was injected with vehicle (saline, 100 μL/100 g body weight, IV), and the other group was injected with tropine (200 μmol/kg, IV). Ventilatory parameters were monitored for 60 min after these injections. The body weights of both groups were similar to one another (*p* > 0.05), therefore, ventilatory parameters related to volumes (e.g., TV, PIF, and PEF) are presented without body weight corrections. The FinePointe (DSI) software constantly corrected digitized ventilatory values originating from actual waveforms for alterations in chamber humidity and chamber temperature. Pressure changes associated with respiratory waveforms were converted to volumes (e.g., TV, PIF, and PEF) using the algorithms of Epstein and Epstein (1978) and Epstein et al. (1980). Factoring in the chamber humidity and temperature, cycle analyzers filtered the acquired signals, and FinePointe algorithms generated an array of box flow data that identified a waveform segment as an acceptable breath. From this array, minimum and maximum box flow values were obtained and multiplied by a compensation factor provided by the selected algorithm, thereby producing TV, PIF, and PEF values used to determine non-eupneic breathing events expressed as non-eupneic breathing index (NEBI, % of non-eupneic breathing events per epoch) (Getsy et al., 2014). Apneic pause was calculated by the formula (Expiratory time/Relaxation time) − 1 (Gaston et al., 2021).

### Protocols for blood-gas measurements and determination of arterial-alveolar gradient

Changes in ABG chemistry values (pH, pCO_2_, pO_2_, and sO_2_) and A-a gradients were determined as detailed previously (Getsy et al., 2022e; f). The A-a gradient defines differences between alveolar and arterial blood O_2_ concentrations (Stein et al., 1995; Story, 1996). For example, a decrease in PaO_2_ without a concomitant alteration in the A-a gradient is the result of hypoventilation, whereas a decrease in PaO_2_ with a concomitant increase in A-a gradient indicates an ongoing mismatch in ventilation–perfusion in alveoli. A-a gradient = PAO_2_ – PaO_2_, where PAO_2_ is the partial pressure (p) of alveolar O_2_, and PaO_2_ is pO_2_ in sampled arterial blood. PAO_2_ = [(FiO_2_ × (P_atm_ – P_H2O_) − (PaCO_2_/respiratory   quotient)], where FiO_2_ is the fraction of O_2_ in inspired air; P_atm_ is atmospheric pressure; P_H2O_ is the partial pressure of H_2_O in inspired air; PaCO_2_ is the pCO_2_ in arterial blood; and respiratory quotient (RQ) is the ratio of (CO_2_ eliminated)/(O_2_ consumed). We took FiO_2_ of room-air to be 21% = 0.21, P_atm_ to be 760 mmHg, and P_H2O_ to be 47 mmHg (Gaston et al., 2021). We took the RQ value of our adult male rats to be 0.9 (Stengel et al., 2010; Chapman et al., 2012). Briefly, on the day of the study, an arterial blood sample (100 μL) was taken from two groups of rats to determine pre-drug (baseline) ABG and A-a gradient values. Thirty minutes later, both groups received a bolus injection of fentanyl (75 μg/kg, IV), and an arterial blood sample (100 μL) was taken post 5 min. Immediately afterward, one group (83.7 ± 0.7 days of age; 339 ± 2 g body weight) received a bolus injection of vehicle, and the other group (83.3 ± 0.9 days of age; 336 ± 2 g body weight) received a bolus injection of tropine (200 μmol/kg, IV). Arterial blood samples were taken from both groups 5 min, 10 min, and 15 min later for determination of ABG values. All ABG chemistry values were determined by a radiometer blood-gas analyzer (ABL800 FLEX).

### Antinociception assessment by tail-flick latency assay

The antinociceptive actions of fentanyl and tropine were determined by tail-flick latencies (TFL) via the use of a Tail-Flick Analgesia Meter (IITC Life Science Inc., United States) as detailed previously (Lewis et al., 1991; Meller et al., 1991; Getsy et al., 2022a; g). This involved a minor degree of manual restraint while positioning the tail to apply a thermal beam sufficient to induce a latency of tail withdrawal of approximately 2.5 s. Baseline TFL were tested in all rats 30–60 min prior to drug injection. Next, all rats received an injection of fentanyl (75 μg/kg, IV), and TFL were recorded at 5 min. One group of rats (83.3 ± 0.9 days of age; 336 ± 1 g body weight, n = 6) then received an injection of vehicle (saline, 100 μL/100 g body weight, IV), and the second group (83.0 ± 0.8 days of age; 335 ± 2 g body weight, n = 6) received a bolus injection of tropine (200 μmol/kg, IV). TFL were then recorded 10 min, 25 min, 40 min, and 55 min after these injections.

### Sedation—righting reflex

Separate groups of rats were used to evaluate the effects of tropine (200 μmol/kg, IV) on the duration of fentanyl (75 μg/kg, IV) impairment of the righting reflex (i.e., the inability to stand on all four legs) as described previously (Jenkins et al., 2021). Each rat was placed in an open plastic chamber to allow the duration of loss of righting reflex to be accurately assessed. The time when the rat spontaneously stood on all four paws and remained so for at least 10 s was taken as the point of recovery of the righting reflex (Ren et al., 2015; 2020; Yu et al., 2018). One group of rats (80.0 ± 0.5 days of age; 333 ± 1 g, n = 9) received an injection of fentanyl and, after 5 min, an injection of vehicle. A second group of rats (79.7 ± 0.4 days of age; 332 ± 1 g, n = 9) received an injection of fentanyl and, after 5 min, an injection of tropine. The duration of the effect of fentanyl was defined as the time interval from the time of injection of fentanyl administration to the recovery of the righting reflex.

### Data analyses

All data are presented as mean ± SEM and were analyzed by one-way and two-way ANOVA with Bonferroni corrections for multiple comparisons between means using the error mean square terms from the ANOVAs (Getsy et al., 2022a—c). A *p* < 0.05 value was taken as the initial significance level and was modified by the number of between-mean comparisons. The modified *t-*statistic for two groups, for instance, is t = (mean group 1 − mean group 2)/[s × (1/n_1_ + 1/n_2_)^1/2^], where s^2^ = mean square within groups term from the ANOVA analysis, and n_1_ and n_2_ are the number of rats in each group. Statistical analyses were done with GraphPad Prism software (*GraphPad Software*, Inc., La Jolla, CA).

## Results

### Ventilatory parameters

**FIGURE 1:**
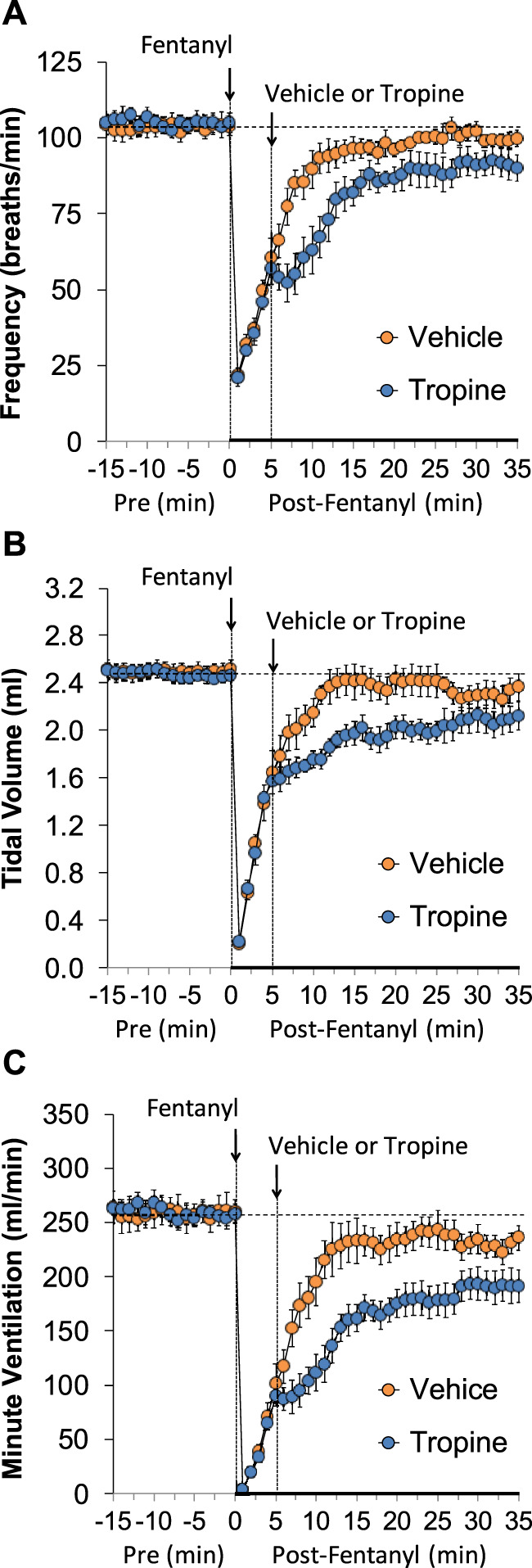
A summary of the values for frequency of breathing **(A)**, tidal volume **(B)**, and minute ventilation **(C)** before (Pre), following injection of fentanyl (75 μg/kg, IV), and subsequent injection of vehicle or tropine (200 μmol/kg, IV) in freely-moving adult male rats. The data are presented as mean ± SEM. There were six rats in each group.

**FIGURE 2:**
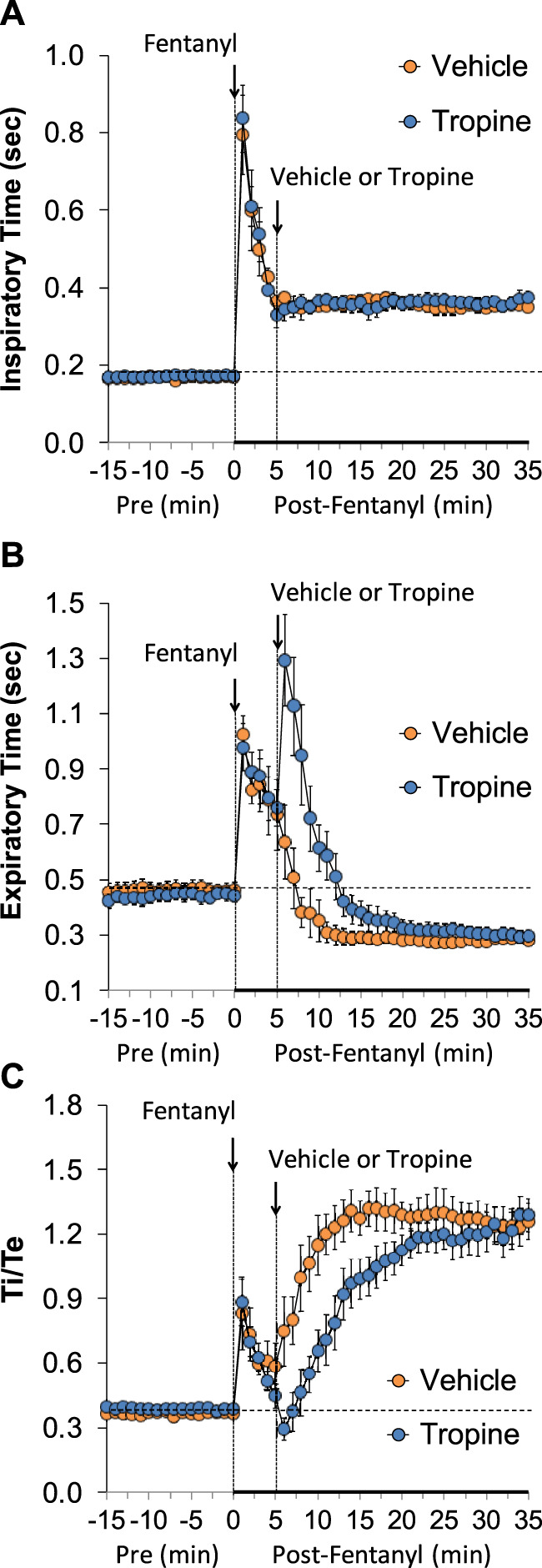
A summary of the values for inspiratory time **(A)**, expiratory time **(B)**, and inspiratory time/expiratory time (Ti/Te) **(C)** before (Pre), following injection of fentanyl (75 μg/kg, IV), and subsequent injection of vehicle or tropine (200 μmol/kg, IV) in freely-moving adult male rats. The data are presented as mean ± SEM. There were six rats in each group.

**FIGURE 3:**
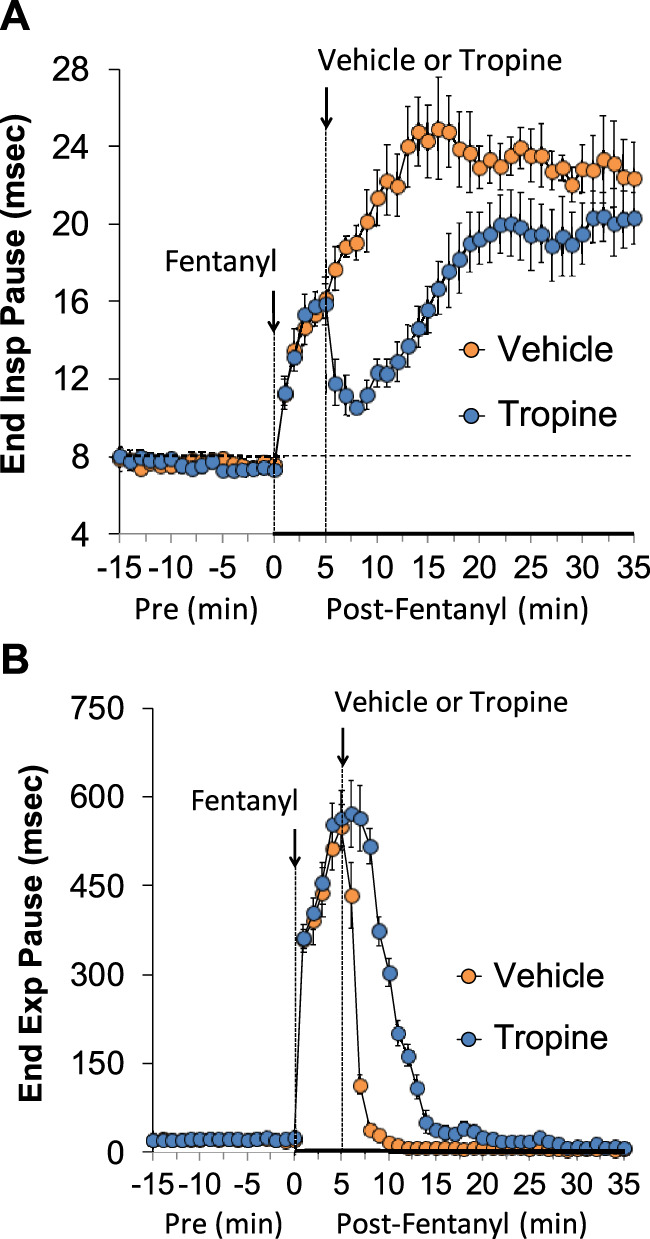
A summary of the values for end-inspiratory pause **(A)** and end-expiratory pause **(B)** before (Pre), following injection of fentanyl (75 μg/kg, IV), and subsequent injection of vehicle or tropine (200 μmol/kg, IV) in freely-moving adult male rats. The data are presented as mean ± SEM. There were six rats in each group.

The ages, body weights, and baseline ventilatory parameters of the two groups of rats used in the ventilatory studies are provided in Supplementary Table S2. There were no between-group differences for any of the ventilatory parameters (*p* > 0.05 for all comparisons). As detailed below, two groups of rats received an injection of fentanyl (75 μg/kg, IV), and, after 5 min, one group received an injection of vehicle (VEH), and the other group received an injection of tropine (200 μmol/kg, IV). Summaries of frequency of breathing (Freq), tidal volume (TV), and minute ventilation (MV) before (Pre), after injection of fentanyl (75 μg/kg, IV), and after subsequent injection of vehicle or tropine (200 μmol/kg, IV) are given in Figure 1. Fentanyl elicited pronounced decreases in Freq (Panel A), TV (Panel B), and, consequently, MV (Panel C) in both groups of rats. Tropine elicited a prompt worsening of Freq, TV, and MV that remained for about 20 min post injection. Values for inspiratory time (Ti), expiratory time (Te), and Ti/Te before (Pre), following injection of fentanyl (75 μg/kg, IV), and after subsequent injection of vehicle or tropine (200 μmol/kg, IV) are summarized in Figure 2. The injection of fentanyl elicited pronounced increases in Ti (Panel A) and Te (Panel B), with the relative changes resulting in brief increases in Ti/Te (Panel C) in both groups. The injection of tropine did not affect Ti, whereas it elicited a prompt increase in Te of about 10 min in duration before declining to similar levels observed in the vehicle-injected rats. As such, tropine markedly diminished the fentanyl-induced increases in Ti/Te. Values for end-inspiratory pause (EIP) and end-expiratory pause (EEP) before (Pre), following the injection of fentanyl (75 μg/kg, IV), and after subsequent injection of vehicle or tropine (200 μmol/kg, IV) are shown in Figure 3. The injection of fentanyl elicited pronounced increases in EIP (Panel A) and EEP (Panel B) in both groups of rats. EIP remained elevated for about 30 min after the injection of vehicle, whereas EEP fell back to baseline within 5 min. The injection of tropine elicited a prompt and relatively sustained decrease in EIP, whereas it lengthened the time of increase in EEP compared to vehicle-injected rats.

**FIGURE 4:**
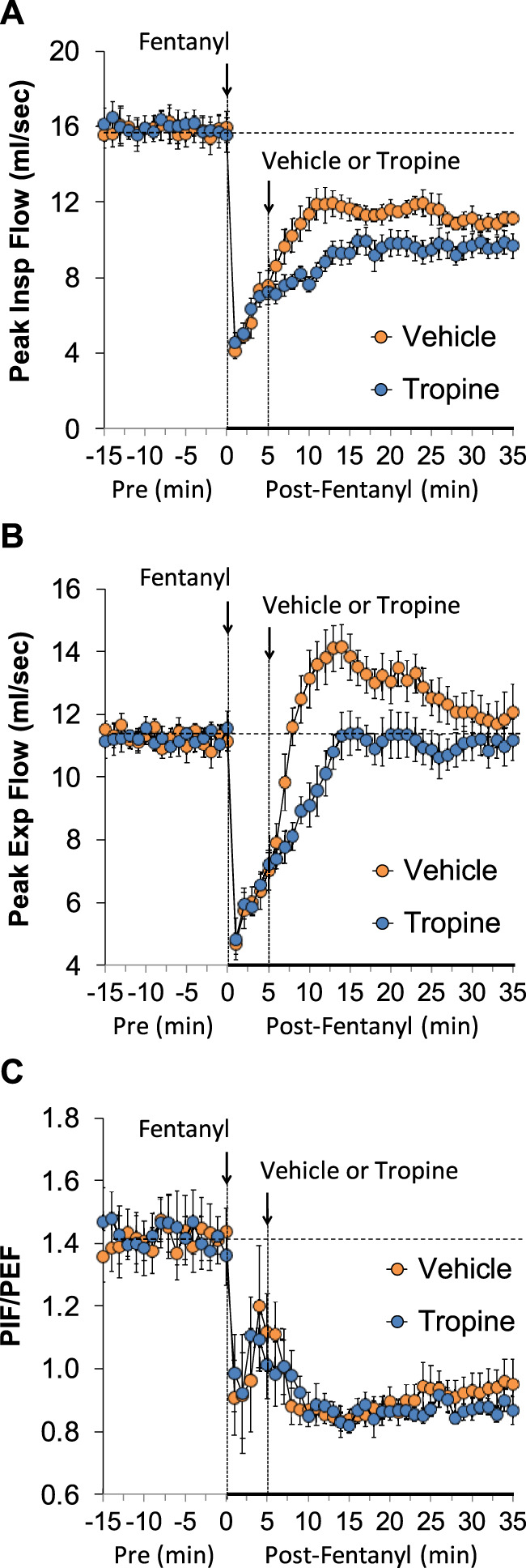
A summary of the values for peak inspiratory flow **(A)**, peak expiratory flow **(B),** and peak inspiratory flow/peak expiratory flow (PIF/PEF) **(C)** before (Pre), following injection of fentanyl (75 μg/kg, IV), and subsequent injection of vehicle or tropine (200 μmol/kg, IV) in freely-moving adult male rats. The data are presented as mean ± SEM. There were six rats in each group.

**FIGURE 5:**
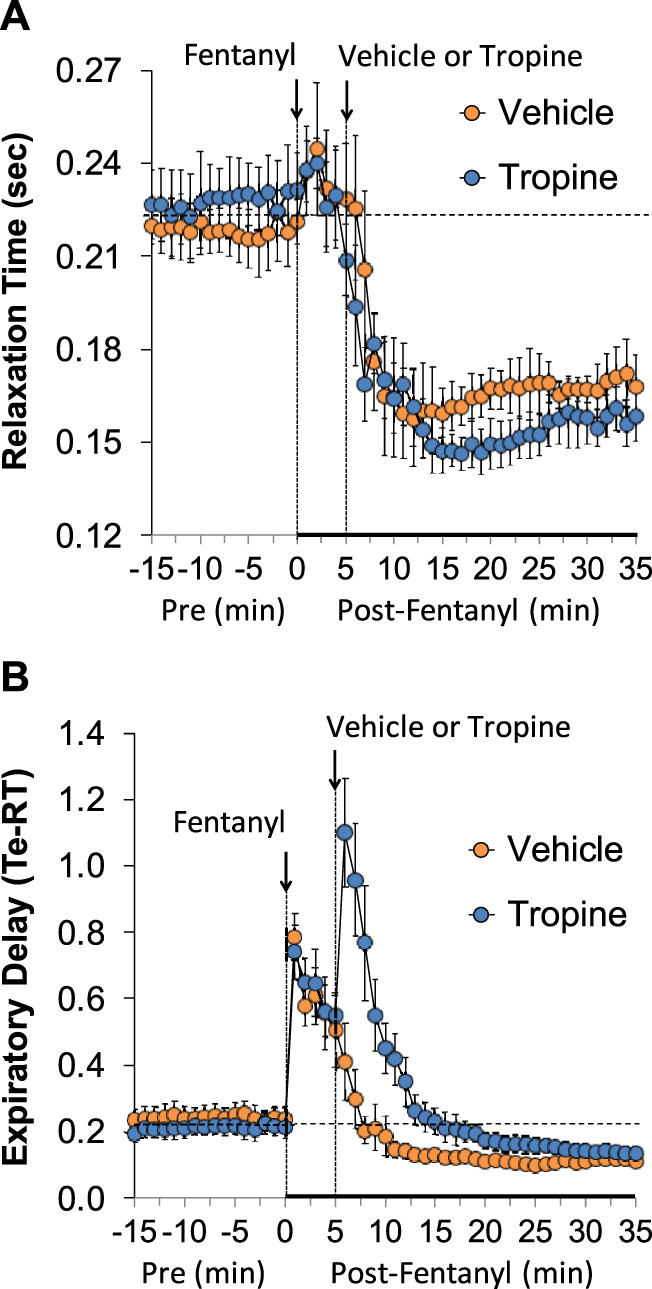
A summary of the values for relaxation time **(A)** and expiratory delay (expiratory time–relaxation time, Te-RT) **(B)** before (Pre), following injection of fentanyl (75 μg/kg, IV), and subsequent injection of vehicle or tropine (200 μmol/kg, IV) in freely-moving adult male rats. The data are presented as mean ± SEM. There were six rats in each group.

**FIGURE 6:**
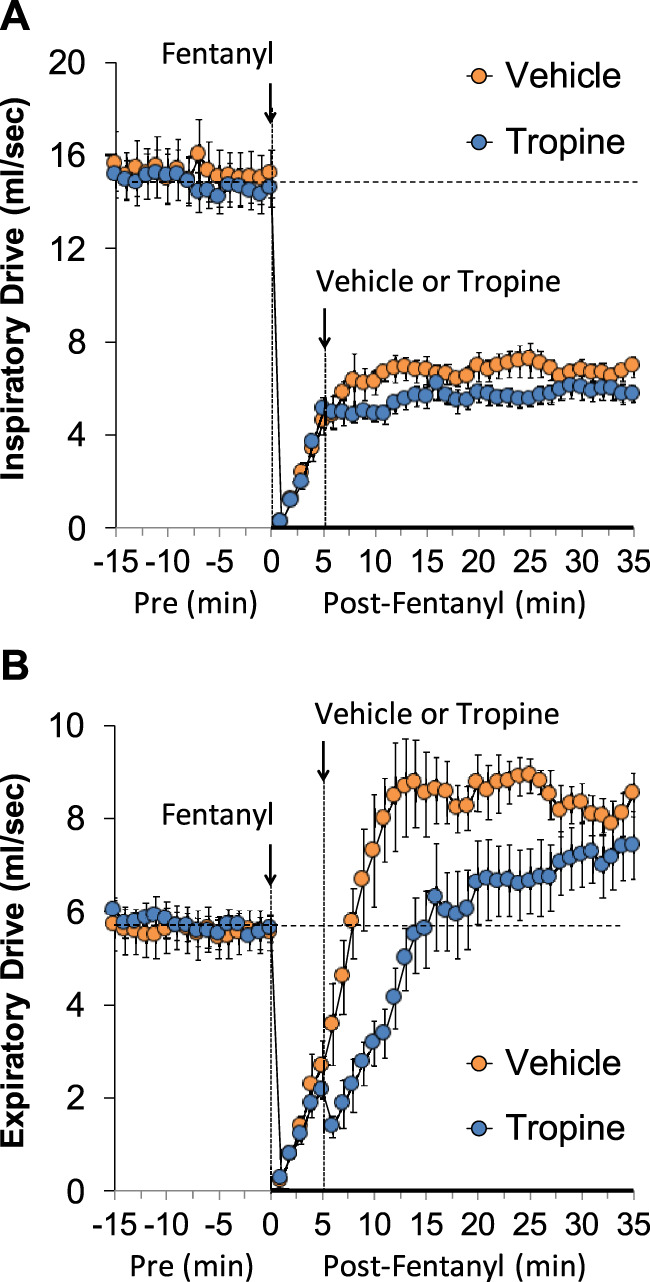
A summary of the values for inspiratory drive **(A)** and expiratory drive **(B)** before (Pre), after injection of fentanyl (75 μg/kg, IV), and subsequent injection of vehicle or tropine (200 μmol/kg, IV) in freely-moving adult male rats. The data are presented as mean ± SEM. There were six rats in each group.

**FIGURE 7:**
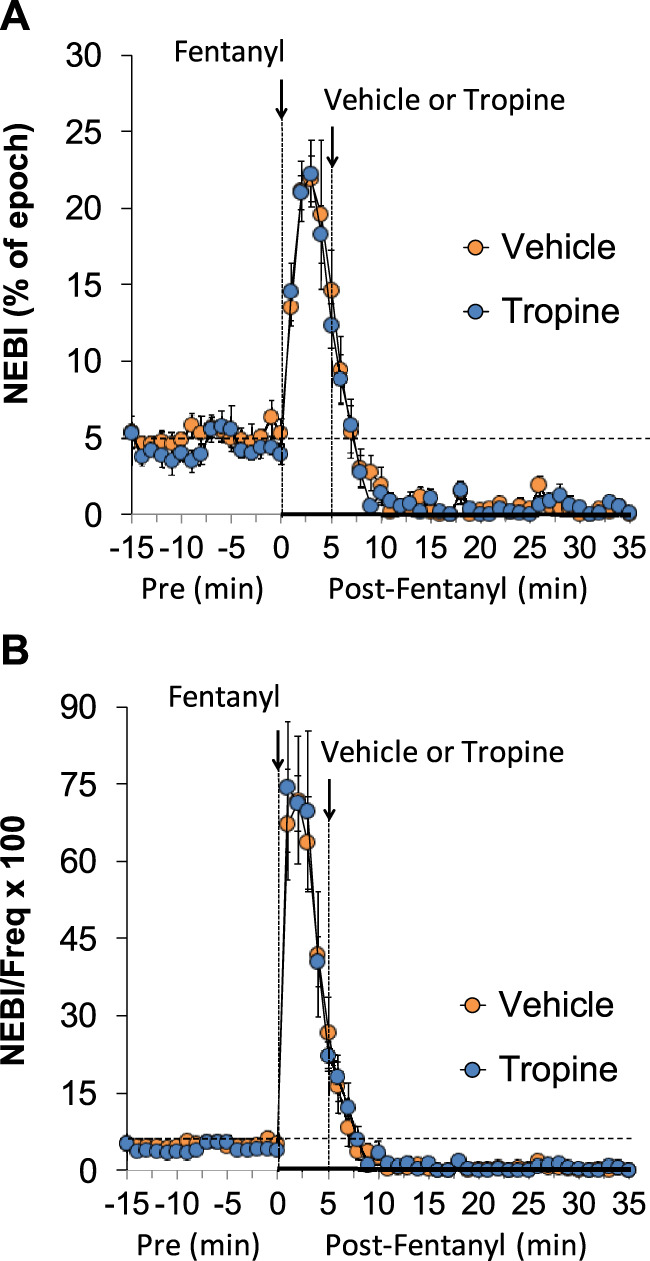
A summary of the values for non-eupneic breathing index (NEBI) **(A)** and NEBI corrected for frequency of breathing (NEBI/Freq) **(B)** before (Pre), following injection of fentanyl (75 μg/kg, IV), and subsequent injection of vehicle or tropine (200 μmol/kg, IV) in freely-moving adult male rats. The data are presented as mean ± SEM. There were six rats in each group.

The values for peak inspiratory flow (PIF), peak expiratory flow (PEF), and PIF/PEF before (Pre), following the injection of fentanyl (75 μg/kg, IV), and after subsequent injection of vehicle or tropine (200 μmol/kg, IV) are summarized in Figure 4. Fentanyl elicited a pronounced and sustained decrease in PIF (Panel A) and a pronounced decrease in PEF (Panel B). PEF levels gradually returned to baseline and then rose above pre-fentanyl values with injection of vehicle (Panel B). The injection of tropine further diminished the fentanyl-induced decrease in PIF and prevented the baseline overshoot in PEF. Taken together, these changes in PIF and PEF resulted in sustained decreases in PIF/PEF that were similar in both groups of rats (Panel C). The values for relaxation time (RT) and expiratory delay (Te-RT) before (Pre), following the injection of fentanyl (75 μg/kg, IV), and after subsequent injection of vehicle or tropine (200 μmol/kg, IV) are summarized in Figure 5. The injection of fentanyl elicited transient increases and then sustained decreases in RT in both groups of rats that were minimally affected by the injection of tropine (Panel A). Fentanyl elicited pronounced increases in expiratory delay that gradually fell to below pre-injection levels in the rats that received an injection of vehicle (Panel B). The injection of tropine caused an immediate and relatively sustained increase in expiratory delay before the values fell to values equal to those of the vehicle-injected rats. The values for inspiratory drive (TV/Ti) and expiratory drive (TV/Te) before (Pre), following the injection of fentanyl (75 μg/kg, IV), and after subsequent injection of vehicle or tropine (200 μmol/kg, IV) are shown in Figure 6. Fentanyl elicited a pronounced and sustained decrease in inspiratory drive (Panel A), and a pronounced decrease in expiratory drive (Panel B) that gradually rose to values above pre-fentanyl injection in vehicle-injected rats. The injection of tropine minimally affected inspiratory drive, but augmented the fentanyl-induced decrease in expiratory drive and thus delayed the subsequent overshoot to above pre-fentanyl levels as seen in the vehicle-injected group. The values for NEBI and NEBI corrected for frequency of breathing (NEBI/Freq) before (Pre), after injection of fentanyl (75 μg/kg, IV), and subsequent injection of vehicle or tropine (200 μmol/kg, IV) are shown in Figure 7. Fentanyl elicited a substantial increase in NEBI (Panel A) and NEBI/Freq (Panel B) that eventually returned to and remained below pre-fentanyl injection values in vehicle-injected rats. The injection of tropine did not affect either parameter.

**FIGURE 8:**
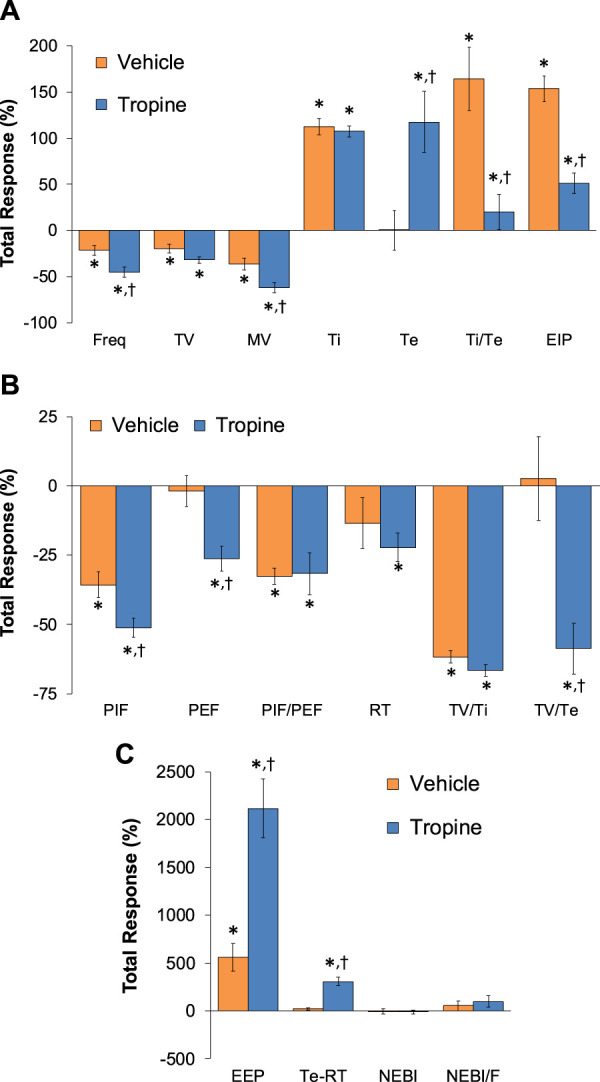
Total (cumulative) changes in ventilatory parameters elicited by fentanyl (75 μg/kg, IV) over the 0–5 min period following the injection of vehicle or tropine (200 μmol/kg, IV). **(A)** Frequency of breathing (Freq), tidal volume (TV), minute ventilation (MV), inspiratory time (Ti), expiratory time (Te), Ti/Te, and end-inspiratory pause (EIP). **(B)** Peak inspiratory flow (PIF), peak expiratory flow (PEF), PIF/PEF, relaxation time (RT), inspiratory drive (TV/Ti), and expiratory drive (TV/Te). **(C)** End-expiratory pause (EEP), expiratory delay (Te-RT), non-eupneic breathing index (NEBI), and NEBI/Freq (NEBI/F). The data are presented as mean ± SEM. There were six rats in each group. *p < 0.05 indicates a significant response from Pre-values. ^**†**^ *p* < 0.05 for tropine *versus* vehicle.

**FIGURE 9:**
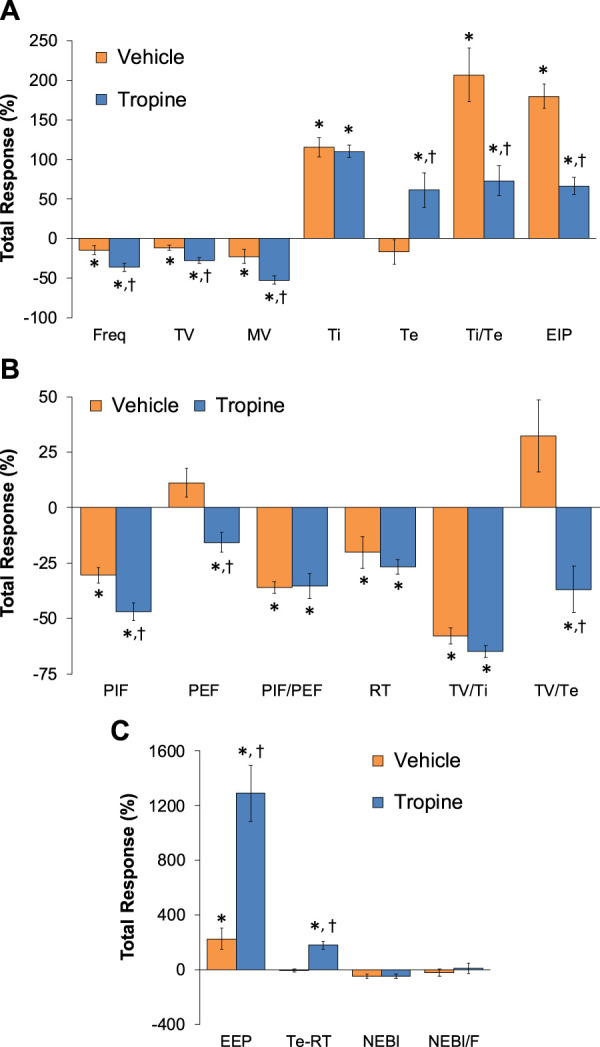
Total (cumulative) changes in ventilatory parameters elicited by fentanyl (75 μg/kg, IV) over the 6–10 min period following injection of vehicle or tropine (200 μmol/kg, IV). **(A)** Frequency of breathing (Freq), tidal volume (TV), minute ventilation (MV), inspiratory time (Ti), expiratory time (Te), Ti/Te, and end-inspiratory pause (EIP). **(B)** Peak inspiratory flow (PIF), peak expiratory flow (PEF), PIF/PEF, relaxation time (RT), inspiratory drive (TV/Ti), and expiratory drive (TV/Te). **(C)** End-expiratory pause (EEP), expiratory delay (Te-RT), non-eupneic breathing index (NEBI), and NEBI/Freq (NEBI/F). Data are presented as mean ± SEM. There were six rats in each group. *p < 0.05 indicates a significant response from Pre-values. ^**†**^ *p* < 0.05 for tropine *versus* vehicle.

**FIGURE 10:**
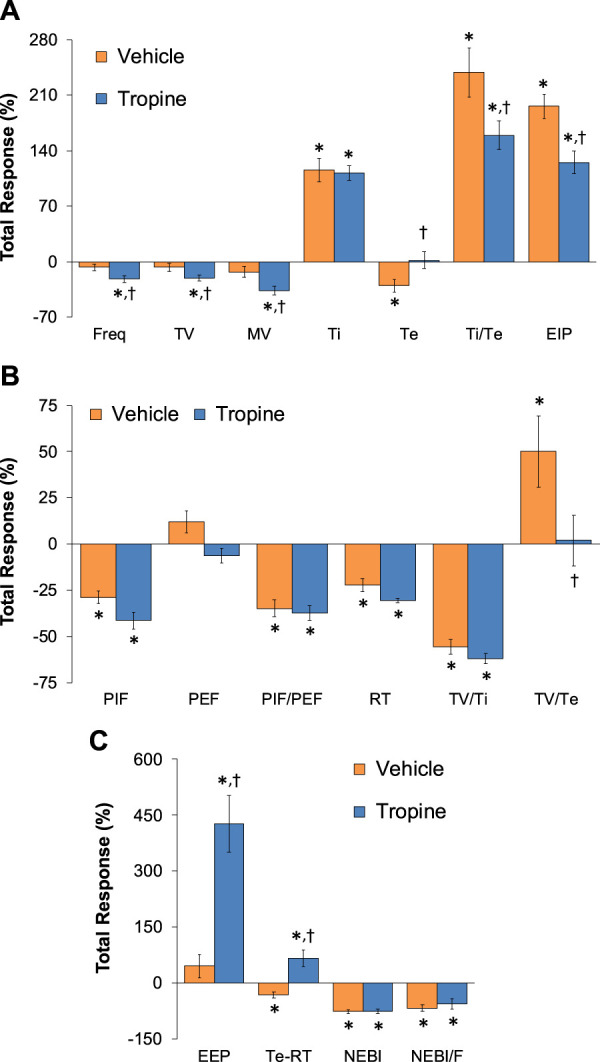
Total (cumulative) changes in ventilatory parameters elicited by fentanyl (75 μg/kg, IV) over the 0–30 min period following injection of vehicle or tropine (200 μmol/kg, IV). **(A)** Frequency of breathing (Freq), tidal volume (TV), minute ventilation (MV), inspiratory time (Ti), expiratory time (Te), Ti/Te, and end-inspiratory pause (EIP). **(B)** Peak inspiratory flow (PIF), peak expiratory flow (PEF), PIF/PEF, relaxation time (RT), inspiratory drive (TV/Ti), and expiratory drive (TV/Te). **(C)** End-expiratory pause (EEP), expiratory delay (Te-RT), non-eupneic breathing index (NEBI), and NEBI/Freq (NEBI/F). Data are shown as mean ± SEM. There were six rats in each group. *p < 0.05 indicates a significant response from Pre-values. ^**†**^ *p* < 0.05 for tropine *versus* vehicle.

The total (cumulative) changes in ventilatory parameters elicited by fentanyl (75 μg/kg, IV) over the 0 to 5 min period prior to injection of vehicle or tropine are summarized in Supplementary Figure S3 **.** Fentanyl elicited significant decreases in Freq, TV, and MV that were associated with increases in Ti, Te, Ti/Te, and EIP (Panel A). Fentanyl elicited substantial decreases in PIF, PEF, PIF/PEF, inspiratory drive (TV/Ti), and expiratory drive (TV/Te), with a substantial increase in expiratory delay (Te-RT) (Panel B). Fentanyl also elicited substantial increases in EEP, NEBI, and NEBI/Freq (Panel C). The total (cumulative) changes in ventilatory parameters over the 0 to 5 min period following injection of vehicle or tropine are summarized in Figure 8. As seen in Panel A, fentanyl elicited decreases in Freq, TV, and MV in vehicle-injected rats that were associated with increases in Ti, Te, Ti/Te, and EIP. The decreases in Freq and MV were greater in tropine-treated rats, whereas the increases in Ti/Te and EIP were diminished in the tropine-treated rats. Additionally, the increases in Te were greater in the tropine-treated rats compared to the vehicle-treated rats. As seen in Panel B, fentanyl elicited substantial decreases in PIF, PIF/PEF, and TV/Ti, but not PEF, RT, or TV/Te in vehicle-treated rats. Tropine-treated rats displayed exaggerated decreases in PIF, PEF, and TV/Te. As seen in Panel C, fentanyl elicited marked increases in EEP, but minimal changes in Te-RT, NEBI, or NEBI/Freq in vehicle-treated rats. The fentanyl-induced increases in EEP and Te-RT were markedly greater in the tropine-treated rats compared to the vehicle-treated rats. The total (cumulative) changes in ventilatory parameters over 6 to 10 min period after the injection of vehicle or tropine are shown in Figure 9. The injection of tropine prevented the effects of fentanyl from fully resolving and instead enhanced the adverse effects of fentanyl on Freq, TV, MV, and Te, while reducing the effects of fentanyl on Ti/Te and EIP (Panel A). Tropine enhanced the adverse effects of fentanyl on PIF, PEF, and TV/Te (Panel B), and EEP and Te-RT (Panel C). The total (overall cumulative) changes in ventilatory parameters over the 0 to 30 min period following the injection of vehicle or tropine are summarized in Figure 10. The effects of fentanyl on Freq, TV, MV, Te, Ti/Te, and EIP (Panel C), TV/Te (Panel B), and EEP and Te-RT (Panel C) were markedly affected by the administration of tropine.

### ABG chemistry and A-a gradient

**FIGURE 11:**
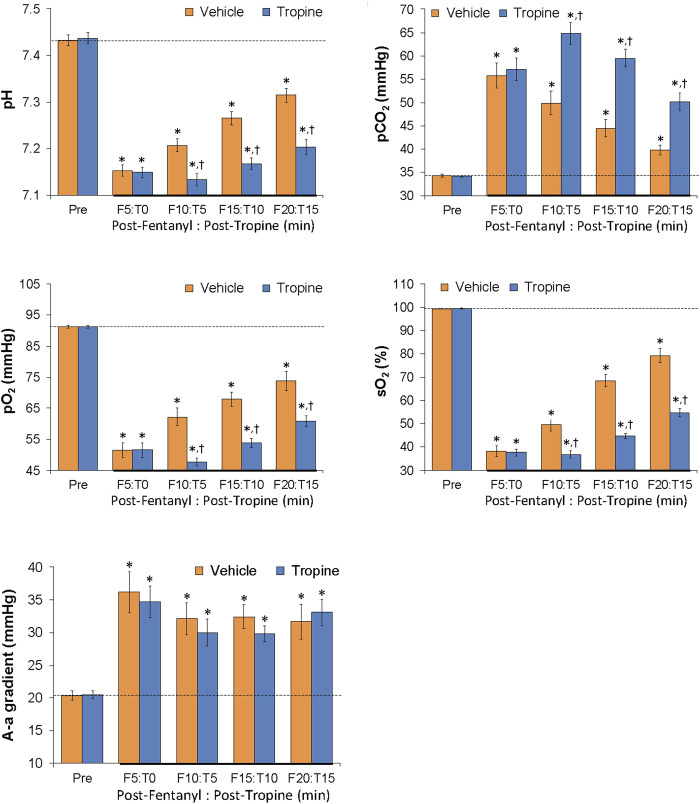
Tropine exacerbates the effects of fentanyl on ABG chemistry and A-a gradient. Values of pH, pCO_2_, pO_2_, sO_2_, and A-a gradient before (Pre) and after injection of fentanyl (75 μg/kg, IV) and then an injection of tropine (200 μmol/kg, IV) given 5 min after fentanyl in freely-moving rats. The data are shown as mean ± SEM. There were six rats in each group. *p < 0.05 indicates a significant change from Pre-values. ^†^ *p* < 0.05 indicates a significant difference from vehicle.

As summarized in Figure 11, the injection of fentanyl (75 μg/kg, IV) elicited substantial decreases in arterial blood pH, pO_2_, and sO_2_ and substantial increases in pCO_2_ and A-a gradient in both groups of rats. All of these deleterious fentanyl responses, except for the increases in A-a gradient, were augmented following the injection of tropine (200 μmol/kg, IV).

### Tail-flick latencies

**FIGURE 12:**
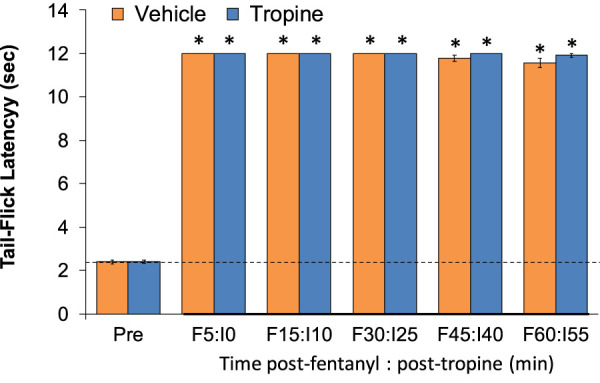
Tropine does not diminish the antinociceptive actions of fentanyl. Tail-flick latency values before (Pre) and after injection of fentanyl (75 μg/kg, IV) and subsequent injections of vehicle or tropine (200 μmol/kg, IV) given 5 min afterward in freely-moving rats. There were six rats in each group. *p < 0.05 indicates a significant change from Pre-values.

As shown in Figure 12, the injection of fentanyl (75 μg/kg, IV) elicited a pronounced antinociception (i.e., increase in tail-flick latencies) that was maintained during the 60-min recording period in rats that received the vehicle injection. Moreover, these antinociceptive effects of fentanyl were identical in magnitude and duration to the vehicle-treated rats in the rats that received tropine (200 μmol/kg, IV).

### Sedation—righting reflex

The injection of fentanyl (75 μg/kg, IV) caused rapid sedative effects in all of the rats studied. More specifically, the rats become immobile with evident chest-wall rigidity. They usually remained on their side with their eyes closed. Full return of the righting reflex (i.e., the ability to stand on four legs) in the tropine (200 μmol/kg, IV)-treated rats (80.1. ± 7.0 min) occurred more slowly than in vehicle-treated rats (57.1 ± 5.6 min) (*p* > 0.05). The injection of tropine did not induce any obvious behavioral differences in the fentanyl-treated rats, except that the fentanyl-induced rigidity appeared to be enhanced, both in strength and duration, in the tropine-treated rats. Upon establishing steady positioning on all four paws, no differences in the normal behaviors of the rats, such as exploring, sniffing, rearing, or grooming, were noted between the vehicle-treated or tropine-treated rats.

## Discussion

This study confirms that the intravenous injection of fentanyl elicits a series of rapid and deleterious effects on ventilatory parameters in unrestrained adult male Sprague Dawley rats (Henderson et al., 2014; Jenkins et al., 2021; Seckler et al., 2022; Getsy et al., 2022a; b). These responses are summarized in Table 1 and described in detail in our companion manuscript (Getsy et al., 2024). These responses included (a) reductions in Freq associated with a sustained elevation of Ti and EIP accompanied by a transient elevation of Te and EEP followed by a sustained reduction in Te and EEP, (b) sustained reductions in TV, MV, and PIF, and more transient decreases in PEF, (c) sustained decreases in RT associated with an initial increase followed by a sustained decrease in expiratory delay (Te-RT), (d) large and sustained decreases in inspiratory drive (TV/Ti) and equally pronounced but more transient decreases in expiratory drive (TV/Te), and (e) substantial increases in NEBI and NEBI/Freq. The ventilatory effects of fentanyl in these rats, including the destabilization of breathing (i.e., increase in NEBI), were expressed when they were heavily sedated and non-moving. As such, it appears that all of these effects of fentanyl were a result of intrinsic mechanisms of action, rather than being caused indirectly by fentanyl-induced behaviors. Several structurally different opioid analgesics, such as fentanyl, morphine oxymorphone, butorphanol, buprenorphine, and methadone, elevate A-a gradients in rats (May et al., 2013a; b; Henderson et al., 2014; Getsy et al., 2022a; b,c; d; e; f; g), goats (Meyer et al., 2006), dogs (Jacobson et al., 1994), rabbits (Shafford and Schadt, 2008), impala (Meyer et al., 2010), and humans (Goetz et al., 1994; Teichtahl et al., 2004; Wang et al., 2005). As was expected, fentanyl elevated the A-a gradient by direct mechanisms involving impairment of alveolar gas exchange or by indirect mechanisms, such as atelectasis (Henderson et al., 2014; Jenkins et al., 2021; Seckler et al., 2022; Getsy et al., 2022a; b). The fentanyl-induced impairment in gas exchange, together with reduced ventilation, resulted in decreases in pH, pO_2_, and sO_2_, and an increase in pCO_2_. The mechanisms responsible for the effects of fentanyl on breathing, alveolar gas exchange, and ABG chemistry involve central and peripheral mechanisms (Henderson et al., 2014). As expected, fentanyl caused robust and long-lasting antinociception and sedation in these rats that has been thoroughly documented and explored (Henderson et al., 2014; Jenkins et al., 2021; Getsy et al., 2022a; b).

**TABLE 1:** Comparison of the effects of Ibutropin and tropine on fentanyl-induced responses.

Parameter	Fentanyl	Ibutropin	Tropine
A. Ventilatory parameters			
Frequency of breathing (Freq)	↓	Reversed	Worsened
Inspiratory time (Ti)	↑	Slight reversal	No effect
Expiratory time (Te)	↑↓	No effect/augment	Worsened/no effect
Ti/Te	↑	Augmentation	Augmentation
End-inspiratory pause (EIP)	↑	Reversed	**Improved/reversed
End expiratory pause (EEP)	↑	Reversed	Worsened
Relaxation time (RT)	↓	Slight augmentation	No effect
Expiratory delay (Te-RT)	↑	Minimal effect	Worsened
Tidal volume (TV)	↓	Reversed/overshoot	Worsened
Minute ventilation (MV)	↓	Reversed/overshoot	Worsened
Peak inspiratory flow (PIF)	↓	Reversed/overshoot	Worsened
Peak expiratory flow (PEF)	↓	Reversal/overshoot	Worsened
PIF/PEF	↓	Slight reversal	No effect
Inspiratory drive (TV/Ti)	↓	Reversed/overshoot	No effect
Expiratory drive (TV/Te)	↓	Reversed/overshoot	Worsened
Non-eupneic breathing index (NEBI)	↑	Reversed	No effect
NEBI/Freq	↑	Reversed	No effect
B. Arterial Blood-Gas Chemistry/Alveolar-arterial Gradient			
pH	↓	Reversed	Worsened
pCO_2_	↑	Reversed	Worsened
pO_2_	↓	Reversed	Worsened
sO_2_	↓	Reversed	Worsened
Alveolar-arterial gradient	↑	Reversed	Worsened
C. Antinociception (tail-flick latency)	↑	No apparent effect	No apparent effect
D. Sedation (Righting reflex)	↑	Shorter duration	Longer duration

This study shows that the injection of tropine augmented the adverse effects of fentanyl on ventilatory parameters in male Sprague Dawley rats and augmented the deleterious changes in A-a gradient and ABG chemistry produced by the synthetic opioid. In addition, tropine increased the duration of fentanyl-induced sedation, but did not alter fentanyl-induced analgesia. The question arises as to where and how tropine exerts these effects on fentanyl. The effects of fentanyl on ventilatory parameters, A-a gradient, ABG chemistry, and analgesia are mediated by central and peripheral opioid receptors (Henderson et al., 2014). As such, the ability of tropine to worsen the effects of fentanyl on ventilation and ABG chemistry may be due to actions in the brain, spinal cord, and peripheral sites (e.g., carotid bodies and chest-wall/diaphragmatic structures). The absence of the effect of tropine on the adverse effects of fentanyl on alveolar gas exchange (A-a gradient) argues against a peripheral site of action of tropine within the lungs. Opioids elevate A-a gradients by impairing ventilation–perfusion ratios in the lungs, known as ventilation–perfusion mismatch (Meyer et al., 2006; Henderson et al., 2014). Opioids diminish pulmonary perfusion via hypoxemia-mediated pulmonary vasoconstriction (Nun, 1993) and by pulmonary vasoconstriction (Santiago and Edelman, 1985; Hakim et al., 1992) via central activation of the sympathetic drive into lung structures (Roquebert and Delgoulet, 1988) and by histamine release in the lungs (Hakim et al., 1992; Mather, 1994). As will be discussed below, Ibutropin may reverse fentanyl-induced increases in A-a gradient via an increase in TV (thereby preventing atelectasis) and/or by modulating the other mechanisms detailed above. The lack of effect of tropine on the A-a gradient suggests that it cannot interact with peripheral and/or central mechanisms by which Ibutropin elicits its effects. In contrast, the ability of tropine to enhance the duration of fentanyl sedation also points to the likelihood that tropine enters and acts within the brain (e.g., cortical) sites responsible for the sedative actions of opioids (Young-McCaughan and Miaskowski, 2001a; b). Importantly, the apparent lack of effect of tropine on the antinociceptive actions of fentanyl suggests that the tropine does not directly block central or peripheral opioid receptors or their cell-signaling pathways that engender analgesia (Henderson et al., 2014; Jenkins et al., 2021). These results suggest that tropine modulates several of the pharmacological effects of fentanyl by actions within the central nervous system and periphery.

As mentioned, our findings with tropine contrast starkly with those obtained with tropine ester, Ibutropin (Getsy et al., 2024). As summarized in Table 1, Ibutropin beneficially reversed and tropine worsened the adverse effects of fentanyl on ventilatory parameters, A-a gradient, and ABG chemistry while not affecting the antinociceptive actions of the opioid. Ibutropin shortened and tropine lengthened the duration of fentanyl-induced sedation. Although sedation is known to be a common effect of opioid analgesics, the mechanisms and behavioral characteristics of sedation are poorly understood (Young-McCaughan and Miaskowski, 2001a; b). However, on the basis of our findings here and in Getsy et al. (2024), we assume that tropine and Ibutropin enter the central nervous system, but exert opposite effects on opioid receptor signaling events. We hypothesize that the effects of Ibutropin involve its ready entry into cells, whereas the effects of tropine may be restricted to modulating functional proteins (e.g., receptors, ion-channels, and enzymes) on the extracellular surface of plasma membranes. Moreover, regarding the different molecular mechanisms of opioid effects on ventilation and antinociception, it has been established that the tropeine-mediated glycine receptor α3-5-HT_1A_–receptor complex initiates subcellular events that overcome fentanyl-induced respiratory depression but not fentanyl-induced analgesia (Manzke et al., 2011). As such, one possibility is that tropine inhibits, whereas Ibutropin activates this receptor complex. Although we do not know whether tropine or Ibutropin differentially modulate the glycine receptor α3-5-HT_1A_-receptor complex or down-stream signaling pathways as do other tropeines (Macksay et al., 2004; 2008; 2009a; b; San Martin et al., 2019; Gallagher et al., 2022), it appears that activation of discrete signaling pathways differentially modulates the ventilatory and analgesic actions of opioids (Manzke et al., 2011). An important difference between Ibutropin and tropine was that Ibutropin, but not tropine, immediately diminished the fentanyl-induced elevation of both NEBI and NEBI/Freq. Considering the evidence as to how opioids affect ventilatory patterns, most non-eupneic breathing events are likely to be apneas, abnormal breaths (mismatch between inspiratory and expiratory phases), and, less often, type 1 and type 2 sighs (Zutler, 2011; Nagappa et al., 2017). The ability of fentanyl to elicit apneas in humans and experimental animals is well established (Willette et al., 1987; Yeadon and Kitchen, 1990; Ren et al., 2009; Zhang et al., 2012a; b; Zhuang et al., 2012; Haouzi et al., 2020; Saunders and Levitt, 2020). The mechanisms by which opioids induce apneas involve opioid receptor-induced signaling events in the Kölliker–Fuse-parabrachial nucleus complex (Saunders and Levitt, 2020), ventrolateral medulla (Willette et al., 1987), and nucleus tractus solitarius (Zhang et al., 2012a; Zhuang et al., 2012). In addition, it has been established that opioids suppress ventilatory responses to hypoxic, hypercapnic, and hypoxic–hypercapnic challenges (Berkenbosch et al., 1997; May et al., 2013a, b). As such, the abilities of fentanyl to depress breathing and elicit apneas may also involve depression of breathing responses to fentanyl-induced alterations in ABG chemistry. The precise sites and mechanisms by which Ibutropin improves the fentanyl-induced increases in NEBI and NEBI/Freq have not been established, but it is evident that tropine cannot mimic the actions of the tropeine. In summary, the processes by which tropine modulates the effects of fentanyl may be multi-factorial and may involve altering the opioid receptor signaling transduction processes triggered by fentanyl acting as a biased-ligand at opioid receptors (Grim et al., 2020a; b). Excitatory–inhibitory interactions between neurons within brainstem respiratory networks provide the basis for steady rhythmic breathing. The rhythmic activity of these networks is maintained by tonic 5HT_1_-receptor-mediated signal transduction processes that maintain synaptic glycine α3 receptors in the active/dephosphorylated state (Manzke et al., 2010). Accordingly, reduced inhibitory glycinergic cell-signaling negatively impacts breathing rhythms (Schmid et al., 1991; Pierrefiche et al., 1998; Busselberg et al., 2001), which may lead to immediate fatalities (Busselberg et al., 2001; Markstahler et al., 2002; Harvey et al., 2008). Moreover, drugs that selectively activate glycine a3 receptors may be beneficial for ventilatory disorders, including those caused by opioids (Lynch et al., 2016). As such, a tropine-mediated inhibition of glycine a3 receptors or their signaling events may be an important factor in the ability of tropine to worsen fentanyl-induced suppression of breathing.

### Study limitations

An important limitation is that we have not performed dose–response relationships with tropine against lower and higher doses of fentanyl in order to maximize our understanding of the efficacy profile of tropine. Additionally, because the effects of opioids can often be quantitatively and qualitatively different in female compared to male rats (Dahan et al., 1998; Sarton et al., 1998; Hosseini et al., 2011), it is imperative to determine the effects of tropine on the pharmacological actions of fentanyl in female rats. It is also imperative to determine whether tropine can prevent the latent deleterious actions of opioids on ventilatory responses elicited by hypoxic–hypercapnic stimuli. Another key limitation of our work is the lack of knowledge about the cellular and molecular mechanisms by which tropine modulates the effects of fentanyl. Based on previous research, we are currently performing receptor binding experiments to establish if tropine directly binds to glycine receptors as do parent tropeine molecules (Macksay et al., 2004; 2008; 2009a; b; Manzke et al., 2010; 2011; San Martin et al., 2019; Gallagher et al., 2022). Finally, we must gather information about the pharmacokinetic profiles of tropine and its potential metabolites in order to better understand the sites of action of these compounds. We are presently modifying our liquid chromatography-mass spectrometry method (Altawallbeh et al., 2019) to establish the distribution of tropine in rats that received the vehicle and those that received fentanyl.

## Conclusion

This study demonstrates that the injection of tropine causes an immediate worsening of the adverse effects produced by fentanyl on ventilatory parameters, alveolar gas exchange (A-a gradient), and ABG chemistry in male Sprague Dawley rats. Additionally, although tropine did not affect fentanyl-induced antinociception, it did lengthen the duration of fentanyl-induced sedation, as assessed by the righting reflex. Taken together, it appears that tropine may not directly block opioid receptors, but rather may modulate the opioid receptor-induced signaling events triggered by fentanyl. Because tropine and Ibutropin have opposing effects on fentanyl-induced changes in ventilatory parameters and ABG chemistry (Getsy et al., 2024), it is possible that the actions of tropine involve interactions with the extracellular domains of functional proteins in plasma membranes, whereas those of Ibutropin may involve interactions with intracellular signaling cascades. The opposing effects of tropine and Ibutropin on the sedative effects of fentanyl suggest that both compounds enter the central nervous system but that the sites and/or mechanisms of action are clearly different. As such, the conversion of Ibutropin to tropine in the body would lead to diminished efficacy of the tropeine. If conversion of Ibutropin to tropine occurs via the hydrolytic actions of blood plasma carboxylesterases, then preventing this hydrolysis by inhibitors, such as bis(4-nitrophenyl) phosphate (Boyce et al., 1976; Butterworth et al., 1993; Nishida et al., 1996), may indeed augment the potency of Ibutropin.

## Data Availability

The raw data supporting the conclusion of this article will be made available by the authors, without undue reservation.
