# Supplementary material for: Tropine exacerbates the ventilatory depressant actions of fentanyl in freely-moving rats
Source: Front Pharmacol. 2024 Jun 24;15:1405461. doi: 10.3389/fphar.2024.1405461 (PMC11228531; doi:10.3389/fphar.2024.1405461)
Supplement: Supplementary file 1 [file DataSheet1.docx]

**Supplementary File**

**Tropine exacerbates the ventilatory depressant actions of fentanyl**

**in freely-moving rats**

Paulina M. Getsy,^1^ Walter J. May,^2^ Alex P. Young,^2^ Santhosh M. Baby,^3,†^ Gregory A. Coffee,^1^ James N. Bates,^4,‡^ Yee-Hsee Hsieh,^5^ Stephen J. Lewis^1,6,7,^*

*^1^Department of Pediatrics, Case Western Reserve University, Cleveland, Ohio, USA*

*^2^Department of Pediatrics, University of Virginia, Charlottesville, Virginia, USA*

*^3^Galleon Pharmaceuticals, Inc., 213 Witmer Road, Horsham, Pennsylvania, USA*

*^4^Department of Anesthesiology, University of Iowa Hospitals and Clinics Iowa, Iowa, USA*

*^5^Division of Pulmonary, Critical Care and Sleep Medicine, University Hospitals Case Medical Center, Case Western Reserve University, Cleveland, Ohio, USA*

*^6^Department of Pharmacology, Case Western Reserve University, Cleveland, Ohio, USA*

*^7^Functional Electrical Stimulation Center, Case Western Reserve University, Cleveland, Ohio, USA*

**^†^Present Address:** Translational Sciences Treatment Discovery, Galvani Bioelectronics, Inc, 1250 S Collegeville Rd, Collegeville, PA 1r9426, USA. Email: babysanthosh@gmail.com

**^‡^Present address:** James N. Bates, Chief Medical Officer, *Atelerix Life Sciences Inc*. Address: 300 East Main Street, Suite 202 Charlottesville, Virginia 22902. Email: jbates@atelerixlifesciences.com. https://atelerixlifesciences.com

***Corresponding Author:** Stephen J. Lewis, PhD. Department of Pediatrics, Division of Pulmonology, Allergy and Immunology, Department of Pharmacology School of Medicine, Biomedical Research Building, Room 831, Case Western Reserve University, 10900 Euclid Avenue, Cleveland, OH 44106-4984. Phone: 843-422-7639. Email: sjl78@case.edu

**Supplementary Figure S1**

**Tropine**

**Ibutropin**


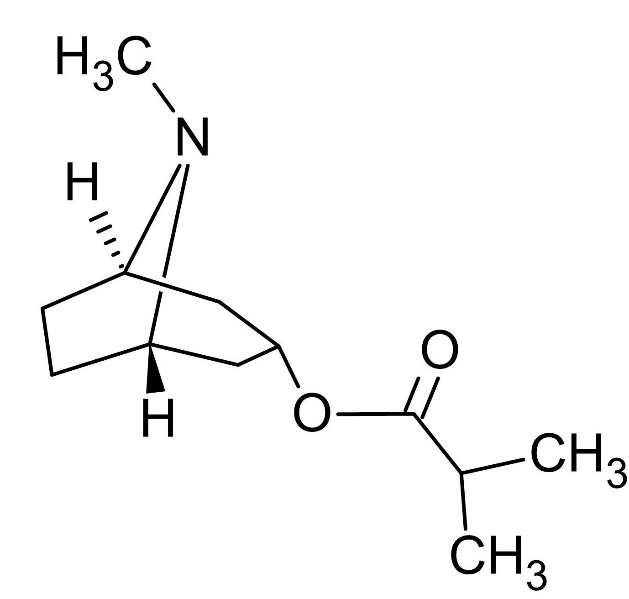


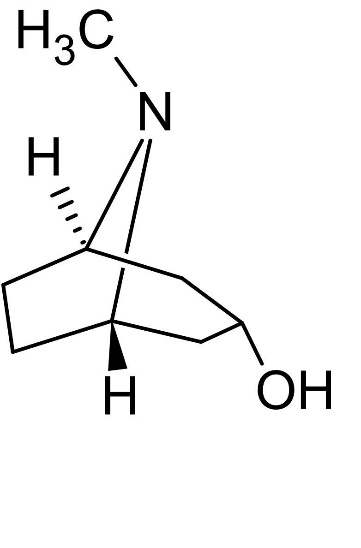


Isobutyric tropine ester; Butropine; Tropine isobutyrate; Iisobutyroyl tropine

[(1R,5S)-8-methyl-8-azabicyclo[3.2.1]octan-3-yl] 2-methylpropanoate

3-tropanol

**Supplementary Table S1**

Definition of ventilatory parameters

| **Parameter** | **Abbreviation** | | **Units** | | **Definition** |
| --- | --- | --- | --- | --- | --- |
| **A. Directly recorded parameters** | | | | | |
| Frequency of breaths | | Freq | | breaths/min | Rate of breathing |
| Inspiratory Time | | Ti | | sec | Duration of inspiration |
| Expiratory Time | | Te | | sec | Duration of expiration |
| End Inspiratory Pause | | EIP | | msec | Pause between end of inspiration and start of expiration |
| End Expiratory Pause | | EEP | | msec | Pause between end of expiration and start of inspiration |
| Relaxation time | | RT | | sec | Decay of expiration to 36% maximum |
| Tidal Volume | | TV | | ml | Volume of inspired air per breath |
| Peak Inspiratory Flow | | PIF | | ml/sec | Maximum inspiratory flow |
| Peak Expiratory Flow | | PEF | | ml/sec | Maximum expiratory flow |
| Expiratory flow at 50% | | EF_50_ | | ml/sec | Expiratory flow at 50% expired TV |
| Non-eupneic breathing index | | NEBI | | % | % of non-eupneic breaths per epoch |
| **B. Derived parameters** | | | | | |
| Minute Ventilation | | MV = freq x TV | | ml/min | Total volume of air inspired per min |
| Ti/Te | | Ti/Te | | none | Inspiratory quotient |
| PEF/PIF | | PEF/PIF | | none | Flow balance |
| Expiratory Delay | | Te-RT | | No units | Difference in lengths of Te and RT |
| Inspiratory Drive | | TV/Ti | | ml/sec | Central urge to inhale |
| Expiratory Drive | | TV/Te | | ml/sec | Central urge to exhale |
| NEBI/Frequency | | NEBI/Freq | | %/(b/min) | Balanced rejection index |

**Supplementary Figure S2**

**Supplementary Figure S2.** Relationships between peak inspiratory flow (PIF), peak expiratory flow (PEF), relaxation time (RT) and expiratory time (Te).

**Supplementary Table S2.** Baseline (Pre) values in the two treatment groups

| **Parameter** | **Pre-Vehicle** | **Pre-Tropine** |
| --- | --- | --- |
| Age, days | 78.7 ± 0.6 | 78.5 ± 0.7 |
| Body weight, grams | 320 ± 2 | 317 ± 2 |
| Frequency, breaths/min | 103.4 ± 3.0 | 105.0 ± 2.6 |
| Tidal Volume, ml | 2.49 ± 0.07 | 2.47 ± 0.04 |
| Minute Volume, ml/min | 258.0 ± 12.7 | 259.4 ± 8.4 |
| Inspiratory Time, sec | 0.168 ± 0.01 | 0.170 ± 0.009 |
| Expiratory Time, sec | 0.462 ± 0.03 | 0.442 ± 0.024 |
| Inspiratory Time/Expiratory Time | 0.37 ± 0.01 | 0.39 ± 0.01 |
| End Inspiratory Pause, msec | 7.7 ± 0.2 | 7.6 ± 0.2 |
| End Expiratory Pause, msec | 22.1 ± 3.3 | 23.2 ± 3.4 |
| Peak Inspiratory Flow, ml/sec | 15.8 ± 0.7 | 15.9 ± 0.7 |
| Peak Expiratory Flow, ml/sec | 11.2 ± 0.2 | 11.2 ± 0.2 |
| Peak Inspiratory Flow/Peak Expiratory Flow | 1.41 ± 0.07 | 1.42 ± 0.07 |
| Relaxation Time, sec | 0.22 ± 0.01 | 0.23 ± 0.01 |
| Expiratory Time – Relaxation Time | 0.24 ± 0.03 | 0.21 ± 0.03 |
| Tidal Volume/Inspiratory Time, ml/sec | 15.2 ± 1.1 | 14.7 ± 0.9 |
| Tidal Volume/Expiratory Time, ml/sec | 5.5 ± 0.5 | 5.7 ± 0.3 |
| Non-Eupneic Breathing Index (NEBI), % | 5.2 ± 0.4 | 4.4 ± 0.1 |
| NEBI(%)/Freq, breaths/min | 5.01 ± 0.45 | 4.18 ± 0.16 |

The data are presented as mean ± SEM. There were 6 rats in each group. There were no between-group differences for any parameter (*p* > 0.05, for all between-group comparisons).

**Supplementary Figure S3**

**A.**

**B.**

**C.**

**Supplementary Figure S3. Fentanyl responses over the 0-5 min period before injection of vehicle or tropine.** Total (cumulative) changes in ventilatory parameters elicited by fentanyl (75 μg/kg, IV) over the 0 to 5 min period prior to injection of vehicle or tropine. **Panel A:** Frequency of breathing (Freq), tidal volume (TV), minute ventilation (MV), inspiratory time (Ti), expiratory time (Te), Ti/Te, and end inspiratory pause (EIP). **Panel B:** Peak inspiratory flow (PIF), peak expiratory flow (PEF), PIF/PEF, relaxation time (RT), expiratory delay (Te-RT), inspiratory drive (TV/Ti), and expiratory drive (TV/Te). **Panel C:** End expiratory pause (EEP), non-eupneic breathing index (NEBI), and NEBI/Freq (NEBI/F). The data are presented as mean ± SEM. There were 6 rats in each group. **p* < 0.05, significant response from Pre-values. There were no between-group differences for any parameter (*p* > 0.05, for all comparisons).
